# Patients’ experiences of using a mobile application-based rehabilitation programme after total hip or knee arthroplasty: a qualitative descriptive study

**DOI:** 10.1186/s12912-023-01409-3

**Published:** 2023-07-27

**Authors:** Qingling Wang, Regina Lai-Tong Lee, Sharyn Hunter, Sally Wai-Chi Chan

**Affiliations:** 1 School of Nursing and Health Management, Shanghai University of Medicine and Health Sciences, Shanghai, China; 2 School of Nursing and Midwifery, College of Health, Medicine and Wellbeing, The University of Newcastle, Callaghan, Newcastle, NSW Australia; 3 The Nethersole School of Nursing, Faculty of Medicine, The Chinese University of Hong Kong, Shatin, Hong Kong SAR, China; 4 Tung Wah College, Homantin, Hong Kong SAR, China

**Keywords:** Mobile application, Patient experience, Qualitative research, Self-efficacy, Total hip arthroplasty, Total knee arthroplasty, Telerehabilitation

## Abstract

**Background:**

An increasing number of patients are discharged from a total hip or knee arthroplasty with a short length of hospital stay. Technologies, such as mobile applications, are used to provide remote support to patients’ postoperative rehabilitation. Patients’ experiences of receiving mobile application-based rehabilitation after total hip or knee arthroplasty have not been investigated extensively.

**Methods:**

This was a qualitative descriptive study. Twenty-five participants who had completed a mobile application-based rehabilitation programme for total hip or knee arthroplasty were recruited. Semi-structured interviews were conducted via telephone between July 2021 and January 2022 regarding the participants’ experiences using the programme. All interviews were audio-recorded and verbatim transcribed. Data were analysed using inductive content analysis. The reporting of this study followed the Consolidated Criteria for Reporting Qualitative Research.

**Results:**

Data analysis revealed five categories: (a) improved access to health care, (b) encouraged postoperative recovery, (c) established supportive relationships, (d) facilitated learning, and (e) future directions.

**Conclusion:**

The theory-underpinned mobile application-based rehabilitation programme demonstrated potential value in supporting patients’ rehabilitation after arthroplasty. Nurses can consider using mobile technologies to expand their role in arthroplasty rehabilitation and improve the quality of rehabilitation care.

**Supplementary Information:**

The online version contains supplementary material available at 10.1186/s12912-023-01409-3.

## Background

Total hip arthroplasty (THA) and total knee arthroplasty (TKA) are two surgeries that are widely performed worldwide [1]. Patients undergoing THA and TKA are often discharged from the acute-care hospital within 4 days after surgery [2]. Due to impairments and functional limitations, such as reduced muscle strength and limited flexibility, rehabilitation is often required after THA and TKA procedures to optimise patients’ clinical outcomes. It involves multidimensional interventions and requires interprofessional teamwork. The International Council of Nurses states that nurses play an important role in all phases (acute, post-acute, and long-term) of rehabilitation [3]. Nurses support patients to improve their body functions and participate in social activities, train patients and caregivers in self-care skills, and provide rehabilitation interventions such as the use of assistive and technological devices; all contribute to empowering patients to live independently and participate in life, thereby enhancing their quality of life [3].

The widely accepted standard care for patients after THA and TKA is facility-based (inpatient or outpatient) rehabilitation that begins shortly after discharge from acute care [4]. Patients attend rehabilitation centres and clinics to receive face-to-face rehabilitation services. However, the services may not be accessible to patients who live in rural or remote areas. For instance, approximately 50% of patients undergoing hip arthroplasty in China did not participate in facility-based face-to-face rehabilitation because they lived far from the hospital or there was a lack of available rehabilitation resources [5]. Domiciliary care is implemented in some countries, such as Canada, to deliver face-to-face rehabilitation services to patients dwelling in the community, but it cannot respond to the rapidly growing demand for services [6]. Unsupervised home-based rehabilitation has become an option in some countries such as the United States of America, Australia, and China [7, 8]. Patient adherence to unsupervised home-based rehabilitation is low due to the lack of healthcare professional support [8].

With technological advancement, information and communication technologies, such as mobile applications (apps), are used by healthcare professionals including nurses to provide remote support to patients’ rehabilitation after THA and TKA. It is referred to as telerehabilitation [9]. Patients discharged from THA and TKA are provided via apps with rehabilitation services, such as exercise demonstrations, reminders, health education, progress monitoring, and follow-up [10]. Previous studies reported such mobile app-based rehabilitation had comparable effects to facility-based or domiciliary care on the outcomes of pain, physical function, range of motion, and health-related quality of life [11, 12].

Despite encouraging findings in research, existing mobile app programmes that target patients undergoing THA and TKA vary significantly in quality, requiring further efforts to improve their content, interactivity, and relevance [13]. The application of relevant theories to mHealth solutions can lead to well-designed interventions and better health outcomes [14]. Bandura’s self-efficacy theory [15] and the Illeris model for learning [16] have been recommended in the development of telehealth solutions [17, 18]. The self-efficacy theory elucidates that an individual’s confidence in their capacity to accomplish a task (e.g., rehabilitation) can be enhanced through four sources: mastery experiences, vicarious experiences, verbal persuasion, and arousal state [15]. The Illeris model suggests promoting a person’s learning process through three dimensions: content, incentive, and interaction [16]. The application of these theories/models helps to develop an interactive programme that engages and motivates patients and improves their adherence to rehabilitation, thereby improving rehabilitation outcomes [17–19].

Patients’ input throughout the design, implementation, and evaluation of a mobile app-based programme also influences the quality of the programme [13]. Patients’ perspectives, such as their experiences of using a programme, are crucial as they may provide important information for successfully implementing the programme [20]. Qualitative interviews are a suitable method to obtain rich information regarding patients’ experiences [21], whereby researchers acquire an in-depth understanding of patients’ perspectives on telerehabilitation. Some studies have interviewed patients after arthroplasty regarding their experiences with telerehabilitation and found that patients were satisfied with the accessibility of services [6] and experienced "closeness at a distance" with therapists as well as competence in exercises under the therapists’ remote guidance [22]. Telerehabilitation programmes improved patients’ engagement, self-management, and motivation in arthroplasty rehabilitation [23]. However, these experiences were derived from patients using telerehabilitation based on videoconferencing [6, 22] or web-based access [23]. Patients’ experiences using a mobile app-based rehabilitation programme remain unknown.

Based on Bandura’s self-efficacy theory [15], the Illeris model for learning [16], and patients’ perceived needs [24], we developed a mobile app-based rehabilitation programme to support patients’ rehabilitation after they were discharged from THA and TKA. In addition to the components included in the existing mobile app-based arthroplasty rehabilitation programmes, such as exercise demonstrations and reminders, the newly developed programme incorporated strategies to enhance patients’ self-efficacy for rehabilitation, such as setting progressive rehabilitation goals and providing stories and short videos from previous patients who had recovered from THA and TKA. Visual presentations with short text messages were used to facilitate patients’ learning of rehabilitation instructions. It is necessary to investigate patients’ experiences using this newly developed programme as the findings can help identify why and how the programme works and how it can be further improved [25]. This is valuable for the continuous development of the present programme and future design of mobile rehabilitation.

## Methods

### Aim

This study aimed to investigate patients’ experiences using a mobile app-based rehabilitation programme that was developed for patients after THA and TKA.

### Design

A qualitative descriptive study was conducted. This research method aims to provide rich and authentic descriptions of participants’ experiences and perceptions of the phenomenon being investigated, whereby researchers can obtain an in-depth understanding of the phenomenon [26, 27]. It is aligned with constructivism, the philosophical perspective adopted in this study, that reality is multiple and subjective as it exists in a variety of dynamic contexts and is perceived differently by different subjects [28]. Qualitative descriptive research emphasises literal descriptions and staying close to the data or “the surface of words and events” [26]. It also requires a subjective interpretation of the data which is supported by verbatim quotations from participants [29]. Qualitative descriptive studies are increasingly employed in nursing and healthcare research to identify why an intervention works or does not work and how the intervention might be improved [30]. The findings are used to achieve quality improvement in practice settings [31]. A qualitative descriptive design is appropriate to explore authentic descriptions of patient users’ experiences with a mobile app-based rehabilitation programme, which will inform the improvement of mobile rehabilitation practice.

This study used semi-structured interviews to collect data. All the interviews were conducted by the first author of this paper. She was a nurse academic who had received training on qualitative research methods and built rapport with the participants through delivering the mobile app-based rehabilitation programme. An interview guide was developed based on a review of the literature exploring patients’ experiences with arthroplasty telerehabilitation [6, 22] and with mobile app-based health support [32]. A panel including three experts in qualitative research and two in rehabilitation care discussed and refined the interview guide to ensure that the questions were relevant to the research aim and understandable to the interviewees. The interview guide consisted of six open-ended questions that covered the topics of satisfaction, support for rehabilitation, perceived benefits and weaknesses, willingness to recommend the programme to others, and suggestions for improvement (Table 1). The reporting of this study followed the Consolidated Criteria for Reporting Qualitative Research [33] (see Additional file 1).

**Table 1:** The guide for semi-structured interviews

No	Questions and probes
1	What is your experience of using the mobile app-based rehabilitation programme? Probe: What features or aspects of the programme do you like/dislike? Could you please tell me the reason for your decision?
2	How do you feel that the mobile app-based rehabilitation programme supports your rehabilitation exercises? Probe: Did the programme change your behaviour related to rehabilitation exercises? Could you please provide any detailed explanations or examples?
3	Apart from rehabilitation exercises, are there any other benefits that you experienced during the use of this programme? Probe: Did this programme affect you in other ways during your recovery?
4	Have you seen any problems, weaknesses, or disadvantages of the mobile app-based rehabilitation programme? Probe: Was it easy to use? Did you have any difficulty using the programme?
5	Are you willing to recommend this programme to your relatives, friends, or neighbours? Why?
6	What suggestions do you have for future improvement of the mobile app-based rehabilitation programme? Probe: How can the programme be improved further so that it would be more useful to you and other patients with the same surgery?

### Participants and settings

To recruit participants who had experienced mobile rehabilitation services, the present study purposefully sampled participants from a pool of participants in a randomised controlled trial (RCT) that used a mobile app-based rehabilitation programme to support patients’ rehabilitation at home after THA and TKA [34]. The study was conducted in a 2,000-bed tertiary hospital in Shanghai, a metropolitan city in eastern China. At the recruitment of the RCT, the first author explained the present study to the potential participants, including the objectives, interview procedures, and voluntary participation. The potential participants indicated on the consent form whether they consented to participate in the qualitative interview. All participants were adult patients (greater than or equal to 18 years) who had undergone a unilateral primary THA or TKA. Those participants who reported in a rehabilitation diary that they had used the programme and completed the required rehabilitation tasks were included in the study. They were able to communicate in Chinese (Mandarin).

### Description of the mobile app-based rehabilitation programme

**Fig. 1:**
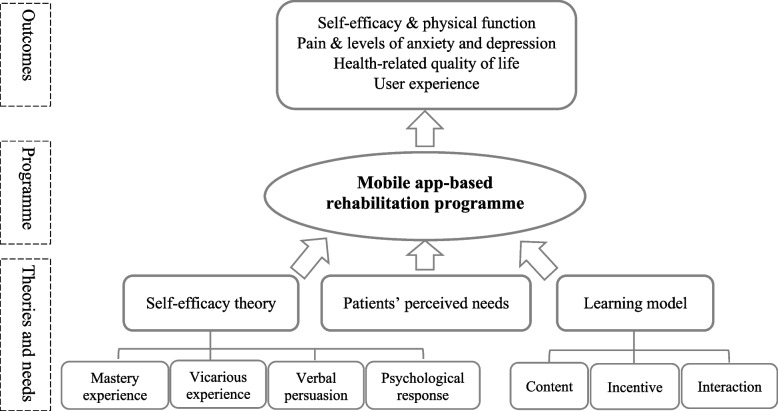
Conceptual framework for a mobile app-based rehabilitation programme

The conceptual framework of the mobile app-based rehabilitation programme is presented in Fig. 1. This programme was based on Bandura’s self-efficacy theory [15], the Illeris model for learning [16], and patients’ perceived needs [24]. It was expected to have positive effects on improving patients’ physical function and self-efficacy, reducing pain and levels of anxiety and depression, and improving patients’ health-related quality of life. A detailed description of the programme is provided in a published paper [34].

The mobile app-based rehabilitation programme was a 6-week programme provided via the app WeChat^®^. It included an exercise regimen aiming to improve patients’ joint mobility, muscle strength, balance, and performance in daily activities. The exercises were demonstrated in videos designed for patients after THA and TKA. Weekly goals for rehabilitation were set, and progressive rehabilitation tasks were scheduled to help the participants achieve their goals, such as gradually increasing walking time from three 5 min per day to 30 min a day. Former patients who had successfully recovered from THA and TKA were invited to share their experiences through stories and short videos. A discussion forum was held via the app so that participants could discuss their conditions with healthcare professionals (i.e., an arthroplasty surgeon, a senior orthopaedic nurse, and a physiotherapist), nursing researchers, and other patients in the programme. Reminders were sent to the participants every Monday to improve engagement. Psychological support measures, such as relaxation exercises and educational materials on depression, were provided via the app. The content of the programme was reviewed by an arthroplasty surgeon, an orthopaedic nurse, a physiotherapist, and two patients. To facilitate patients’ learning via the app, educational materials were provided in visual presentations with short text messages, and all content on the app was ensured to be comprehended by a sixth grader. Each participant was provided with a booklet that illustrated how to use the programme. The educational materials (e.g., demonstration videos) were uploaded to the app, and the participants could learn via WeChat on their own mobile devices. The participants were advised to complete the rehabilitation tasks at least 5 days a week for 6 weeks. They could record their use of the programme and completion of the rehabilitation tasks in a written rehabilitation diary.

### Data collection

Data collection occurred between July 2021 and January 2022. The first author contacted the participants within a week of completing the interventions of the RCT. The study objectives were explained again, and an appointment was made for a telephone interview that would be conducted in Chinese. Data saturation was used to determine the sample size for this study. Saturation occurred when no new codes and/or categories were identified in the data from additional interviews [35]. Data saturation was reached at the 22^nd^ interview, confirmed by no new codes being identified in three more interviews. The interviews ranged from 13 to 29 (mean = 19) minutes.

The interview started with restating the procedure and participants’ rights. Then questions were asked within the boundary of the interview guide. When the participants were speaking, the researcher listened carefully and respectfully with the least interruption. The researcher used reflective approaches, such as making reflection statements (e.g., Can you tell me more about that?) to facilitate the interview process. At the end of each interview, the researcher confirmed with the participant that they had no additional comments and enquired if they could be contacted for further information. All interviews were audio recorded. The first author verbatim transcribed the record in its original language immediately after the interview. Field notes were made to record the researcher’s reflective thoughts.

### Data analysis

Participant demographics were summarised using frequencies and percentages. Inductive content analysis was used for interview data. Instead of preconceived categories, inductive content analysis derived categories and names of categories from the text data [36]. This approach is recommended when previous knowledge about the phenomenon under investigation is inadequate or fragmented [37]. The data analysis followed a three-phase method developed by Elo and Kyngas [37].

- Preparation phase: The first author read the transcripts repeatedly to achieve data immersion. It was followed by reading the data line by line and highlighting the text words that appeared to respond to the research questions.
- Organising phase: The first author noted down in the margins of the transcript her preliminary analysis of the description of the content. These notes were collected onto a coding sheet to create an initial coding scheme. These initial codes were sorted into groups by comparing the similarities and differences between codes. The grouped codes were listed and further grouped into subcategories and then categories, based on the relation and links. Finally, the categories, sub-categories, and codes were refined, and a general description of the research topic was formulated. Throughout the organising process, all authors of this article, including three bilinguals who are fluent in Mandarin and write Chinese, discussed coding and codes, generation of categories/subcategories, and interpretation of findings on a weekly basis.
- Reporting phase: The categories, subcategories, and illustrative quotes were translated from Chinese to English. All authors examined and reported the findings. Discrepancies were discussed by all authors until a consensus was reached.

### Ethical considerations

This study was approved by the Human Research Ethics Committee of the University (No: H-2021–0414) and the Ethics Committee of the hospital (No: B2021-096R). All participants were informed that they voluntarily participated in this study and could withdraw at any time without a consequence. Identification code numbers were used in data collection and analysis to ensure participants’ privacy and anonymity. Collected data were saved in a password-protected cloud system of the university and only accessible to research team members. Written informed consent was obtained for the interviews and audio recording of the interviews.

### Rigour

The strategies described in Table 2 were employed to enhance the trustworthiness of qualitative data [38, 39]. Purposeful sampling and semi-structured interviews were employed to gain insights into the experiences of patients using the mobile app-based rehabilitation programme. The first author who had a prolonged engagement with the participants conducted all interviews using an interview guide to ensure similar questions were asked to all participants. The researcher was aware that the relationship she had built with the participants during the delivery of the mobile app-based rehabilitation programme, as well as her previous experiences in clinical work and research, might bring her a position of power in interviews and preconceptions in data analysis. She tried to bracket her subjectivity from the study by adopting strategies such as continuously recording reflective thoughts in field notes and research memos, listening attentively and respectively during interviews, and analysing the data authentically with an open mind [40, 41]. Further, data and findings were reviewed and discussed repeatedly by all authors including three bilinguals (English/Chinese) to reach a consensus. No participants reviewed the transcript of their interview although they were provided with this option. Thick descriptions and illustrative quotes were presented to support the findings. An audit trail was established by documenting in detail key research stages (e.g., development of an interview guide) and the evolvement of researchers’ thoughts (e.g., research memos).

**Table 2:** Strategies for enhancing trustworthiness

Criteria	Strategies
Credibility	• Adopted purposeful sampling to ensure information-rich cases were selected;
	• Had a prolonged engagement with study participants to build trust and rapport and to understand the context;
	• Used semi-structured interviews to allow participants to freely express their perspectives;
	• Used data saturation to ensure adequate data were collected;
	• Read data repeatedly to obtain immersion; refined and revised codes, subcategories, and categories until obtaining an intended depth of insight
Dependability	• Used an interview guide to ensure similar questions were asked to all participants;
	• Transcribed verbatim immediately after each interview session;
	• Discussed regularly within the research team data collection and analysis
Confirmability	• Set up an audit trail by documenting key research stages (e.g., development of an interview guide, sample selection, transcripts, and draft reports) and evolvement of researchers’ thoughts (e.g., memos of data analysis and reflective thoughts);
	• Provided participants with the option to review the transcript of their interview
Transferability	• Provided thick descriptions of the study context, participant characteristics, and the process of data collection and analysis;
	• Reported the findings together with illustrative quotes from participants
Reflexivity	• Recorded reflective thoughts in field notes and research memos;
	• Listened attentively and respectively during interviews;
	• Analysed the data authentically with an open mind

## Results

This study interviewed 25 participants, 68% of whom were women (*n* = 17). Their age ranged from 33 to 83 (median = 68, interquartile range = 10) years. The participants had undergone TKA (*n* = 10, 40%) or THA (*n* = 15, 60%) procedures, and osteoarthritis was the most common reason for surgery (*n* = 16, 64%). Most participants lived with family (*n* = 22, 88%). The education level of the participants varied: tertiary (*n* = 9, 36%), secondary (*n* = 13, 52%), primary (*n* = 2, 8%), and no education (*n* = 1, 4%). Many participants (*n* = 20, 80%) had concomitant diseases, and hypertension (*n* = 14, 56%) was the most common. Table 3 presents the participants’ demographic characteristics.

**Table 3:** Participants’ demographics and clinical information

ID code	Gender	Age (year)	Residence	Co-habitation	Education level	Work status	Surgery	Reason for surgery	Concomitant disease
P3	Male	68	Suburban	Lives with family	Tertiary	Retired	TKA	Osteoarthritis	Diabetes mellitus
P5	Male	77	Urban	Lives with family	Tertiary	Retired	THA	Osteoarthritis	Hypertension, Prostatitis
P15	Male	33	Suburban	Lives with family	Tertiary	Paid worker	THA	FHAN	SLE
P22	Female	64	Urban	Lives with family	Secondary	Retired	THA	Osteoarthritis	Hypertension
P26	Female	44	Urban	Lives with family	Secondary	Paid worker	THA	DDH	Hypertension, Depression
P27	Male	81	Urban	Lives with family	Tertiary	Retired	TKA	Osteoarthritis	Hypertension, Gout, Atrial fibrillation
P28	Female	47	Other provinces	Lives with family	Secondary	Paid worker	THA	FHAN	None
P31	Female	68	Urban	Lives with family	Tertiary	Retired	TKA	Osteoarthritis	Hypertension
P32	Female	82	Suburban	Lives with family	Tertiary	Retired	THA	Osteoarthritis	Hypertension, Diabetes mellitus
P35	Female	57	Other provinces	Lives with family	Tertiary	Retired	THA	DDH	None
P43	Female	68	Other provinces	Lives with family	No education	Housewife	THA	Fracture	None
P51	Male	64	Urban	Lives with family	Tertiary	Retired	THA	Fracture	None
P54	Female	79	Urban	Lives with family	Secondary	Retired	THA	Fracture	Hypertension
P58	Male	69	Other provinces	Lives with family	Secondary	Retired	TKA	Osteoarthritis	Diabetes mellitus
P60	Female	69	Suburban	Lives with family	Secondary	Retired	TKA	Osteoarthritis	Hypertension
P61	Male	74	Urban	Lives with family	Tertiary	Retired	TKA	Osteoarthritis	Hypertension
P62	Female	65	Suburban	Lives with family	Secondary	Retired	TKA	Osteoarthritis	Hypertension
P64	Female	64	Other provinces	Lives with family	Secondary	Retired	THA	Osteoarthritis	None
P66	Female	63	Urban	Lives alone	Secondary	Retired	TKA	Osteoarthritis	Hypertension, Hyperlipidaemia
P69	Male	73	Suburban	Lives with family	Secondary	Retired	THA	Fracture	Diabetes mellitus
P71	Female	74	Urban	Lives with family	Primary	Retired	THA	Osteoarthritis	Hypertension, Herniated disc
P72	Female	55	Suburban	Lives alone	Secondary	Retired	TKA	Osteoarthritis	Hyperlipidaemia
P74	Female	67	Suburban	Lives with family	Secondary	Retired	THA	DDH	Sjogren's syndrome
P75	Female	80	Suburban	Lives alone	Secondary	Retired	THA	Osteoarthritis	Hypertension
P78	Female	83	Urban	Lives with family	Primary	Retired	TKA	Osteoarthritis	Hypertension

*Abbreviations: TKA* Total knee arthroplasty, *THA* Total hip arthroplasty, *FHAN* Femoral Head Avascular Necrosis, *SLE* Systemic Lupus Erythematosus, *DDH* Developmental dysplasia of the hip

All participants described positive experiences using the mobile app-based rehabilitation programme. Data analysis revealed five categories: (a) improved access to health care, (b) encouraged postoperative recovery, (c) established supportive relationships, (d) facilitated learning, and (e) future directions. Table 4 presents the categories and subcategories revealed in the data analysis.

**Table 4:** Summary of categories and subcategories

No	Categories	Subcategories
1	Improved access to health care	1. Accessed more comprehensive and reliable rehabilitation information
		2. Better access to healthcare professionals
		3. Access to rehabilitation was convenient and flexible
2	Encouraged postoperative recovery	4. Increased engagement in recovery
		5. Increased motivation in rehabilitation exercise
		6. Enhanced confidence for rehabilitation
3	Established supportive relationships	7. With healthcare professionals
		8. With other patients in the programme
4	Facilitated learning	9. Facilitated understanding
		10. Facilitated memorisation
5	Future directions	11. Future use
		12. Suggested programme improvement

### Improved access to health care

All participants described that the mobile app-based rehabilitation programme improved their access to health care. Three subcategories described the improvement.

#### Accessed more comprehensive and reliable rehabilitation information

Most participants described increased access to rehabilitation information through using the mobile app-based rehabilitation programme. The participants said that compared to the pamphlets provided by ward nurses, the information provided in the programme was more comprehensive, encompassing exercise demonstration videos, postoperative precautions, and other relevant content such as relaxation exercises.

*“The programme provided video demonstrations for all exercise movements that I was told to do for rehabilitation. ... I had read the instructional pamphlet provided by the ward nurse, but I preferred to use the app as the information provided via the app was more comprehensive, including the precautions after the surgery and relaxation exercises that could be done after surgery to help relieve stress.” (P5, male, THA)*

The participants said they considered the rehabilitation information provided in the programme more reliable than that they searched on the Internet, and they did not need to judge what were useful suggestions from a huge amount of information.

*“If I had not participated in the programme, I might search for information on the Internet, which might not be accurate, and it would make me confused as the Internet contained many different doctors’ statements and patients’ stories. I cannot tell which one is useful. The information provided in the programme is more reliable.” (P15, male, THA)*

Some participants said that increased access to rehabilitation information via the app had helped them better understand their conditions and have a realistic perception of their postoperative recovery process.

*“The programme helped me to have a realistic perception of the recovery process. ... With the information in the programme, I better understood my conditions. I understood I needed to be more patient when sometimes I felt a bit of discomfort in this (operated) leg, as it was in the recovery process.” (P58, male, TKA)*

However, a few participants said that they did not need the information related to psychological support, such as relaxation exercises, as they viewed such information as only for patients who had psychological problems.

*“I did not need to read the materials in the module on psychological support because I was fine and I had no psychological problems. … I thought they (educational materials regarding psychological support) were only for people who had the problems.” (P5, male, THA)*

#### Better access to healthcare professionals

Many participants described they required support from healthcare professionals in the first few weeks after surgery. They said that the mobile app-based rehabilitation programme, which commenced immediately after hospital discharge, enabled them to better access healthcare professionals and obtain timely support, such as seeking health consultations.

*“During the first few weeks postoperatively, I had many questions about precautions, exercises, self-care, and so on. By using the programme, I was able to obtain support from the healthcare professionals, such as seeking consultations immediately after I was discharged from the hospital. I felt like I had better access to healthcare professionals for support than those who were discharged from the hospital without the mobile app programme.” (P66, female, TKA)*

Some participants said they appreciated that the mobile app-based rehabilitation programme allowed them to seek individual consultations with healthcare professionals. The participants sent messages via the app for consultations about their condition, and the healthcare professionals replied individually through messaging.

*“I went to buy some groceries the third day after hospital discharge, and my operated leg was swollen severely after I came back home. I was scared so I sent a message via the app to ask about it. The healthcare professional responded quickly, messaging me to lie down and exercise on the bed and to reduce walking in the following two days. The swelling disappeared by the third day and never occurred again. I appreciated this programme as I had advice about my condition from the healthcare professionals when I needed it.” (P22, female, THA)*

Other participants described posting questions in the discussion forum for health consultations. The healthcare professionals’ responses and patients’ follow-up feedback were also posted in the discussion forum. The participants said they posted their response to the healthcare professionals’ advice because they thought it might be helpful to other patients in the programme who had a similar issue.

*“Initially, I could not roll over in bed as I felt intense pain in my operated leg when I tried to do so. I asked about it in the discussion forum via the app. The healthcare professional suggested I roll over with the operated leg flexing a bit. It did work, so I posted my feedback in the forum as well because I thought the information might help others who had the same problem.” (P26, female, THA)*

Several participants said the mobile app-based rehabilitation programme had special value during the COVID pandemic, as it allowed patients to obtain support from healthcare professionals while having fewer worries about being exposed to the virus.

*“It is of special value for patients to use the mobile programme for rehabilitation during the COVID-19 pandemic. We could access and obtain support from healthcare professionals without visiting the hospital. This would reduce the need of going to places where social distancing is difficult, such as buses, and thus reduce my chances of being exposed to the virus.” (P60, female, TKA)*

#### Access to rehabilitation was convenient and flexible

Many participants said using the mobile app-based rehabilitation programme was convenient as the mobile phone was a device that they used every day. The programme was provided via the mobile app WeChat, which they were familiar with and had no difficulty using. This made the programme the first choice for them to seek help when they had questions about rehabilitation.

*“Nowadays people are always having a mobile phone in their hands. Especially, I was resting at home after the surgery and had nothing else to do. I held the mobile phone in my hand for almost the whole day. It was very convenient for me to read something on the app.” (P28, female, THA)*

*“Most people of my age are familiar with WeChat as we use it every day. I have no difficulty using it. … When I felt something was wrong while I was doing the exercise, for example, I felt bad in the exercise Clamshell, the first thing I thought was to ask about it via the app.” (P35, female, THA)*

The participants described that using the mobile app-based rehabilitation programme was more convenient than attending rehabilitation centres. They said they were able to learn and practise rehabilitation exercises without worrying about transport and transport-related issues, such as traffic jams and parking.

*“I did not need to go somewhere, like the rehabilitation centre, to learn how to conduct rehabilitation exercises. Traffic and parking issues always give me headaches. I just stayed at my home, watching the app and practising rehabilitation exercises with the support of the mobile app programme.” (P26, female, THA)*

Some participants said that compared to the usual care provided by the hospital (i.e., verbal explanation of the rehabilitation regimen at hospital discharge, a pamphlet to take home illustrating rehabilitation exercises, and being able to contact ward nurses during working hours), they preferred to use the mobile app-based rehabilitation programme because they could flexibly access educational materials without time and place restrictions.

*“I liked to use this programme, without bothering to call nurses or doctors, which could only be done during work time. I just read the educational materials whenever and wherever I wanted and got knowledge about my recovery. I like this kind of flexibility.” (P72, female, TKA)*

### Encouraged postoperative recovery

Sixty per cent (15 out of 25) participants said that participating in the mobile app-based rehabilitation programme encouraged them in postoperative recovery. There were three subcategories.

#### Increased engagement in recovery

The participants described valuing the discussion forum that was held via WeChat. They said that participating in the discussion forum increased their engagement in postoperative recovery as questions and discussions were frequently posted in the forum, which reminded them of practising rehabilitation exercises.

*“I browsed the posts on the discussion forum twice a day during my rehabilitation. People posted questions, and the healthcare professionals provided answers quickly. The posting activities constantly reminded me of my rehabilitation. I would not forget to practise rehabilitation exercises, and I took post-surgery precautions. I felt I was more engaged in my recovery when participating in the discussion forum.” (P3, male, TKA)*

The participants valued the messages that were sent to them every Monday reminding them to continue rehabilitation exercises. They said these reminders helped them in undertaking rehabilitation exercises.

*“The healthcare professionals sent messages every Monday to remind me to continue exercising, even during the holiday. With these reminders, I did better at keeping going with the practice of rehabilitation exercises.” (P64, female, THA)*

The participants described their communication with healthcare professionals via the mobile app programme. They said timely communication with healthcare professionals engaged them more in the management of their postoperative recovery.

*“I was wondering why my right leg was numb, so I sent a message in the evening to ask about it. The healthcare professional responded immediately, messaging me to explain the issue and encourage me to keep observation of my symptom. Through timely communication with the healthcare professional, I felt engaged to do more for my recovery.” (P22, female, THA)*

#### Increased motivation in rehabilitation exercise

Some participants described being motivated to conduct rehabilitation exercises by using the components of the mobile app-based rehabilitation programme, such as the exercise demonstration videos and the discussion forum. They said that practising following video demonstrations and support from healthcare professionals and other patients motivated them to become more active in their rehabilitation exercises.

*“In the beginning, I struggled to keep up with the video demonstrations provided in the mobile programme. After some time, following the demonstrator was a piece of cake. I felt motivated in the conduction of rehabilitation exercises, and gradually I did some extra exercises according to my condition.” (P74, female, THA)*

*“If I had any questions about rehabilitation exercises, I could ask any time in the discussion forum, and the healthcare professionals and other patients would help me. Their support motivated me to be more active in rehabilitation practice as I had no worries about the exercises.” (P54, female, THA)*

One participant described her experience compared with other patients who were not provided with the mobile app-based rehabilitation programme. She said she appreciated the support provided by the healthcare professionals in the programme, and this appreciation motivated her to work harder on rehabilitation exercises.

*“One of my friends underwent the same surgery at a local hospital, and she was only provided with paper instructions on rehabilitation exercises. She asked me to share with her the demonstration videos because she had some difficulties understanding the paper instructions. Compared to her, I felt I was lucky because I was able to learn many materials besides demonstration videos in this mobile programme. I worked harder on rehabilitation exercises as I appreciated the support that healthcare professionals had provided for my postoperative recovery.” (P35, female, THA)*

#### Enhanced confidence for rehabilitation

Many participants described their confidence in mastering rehabilitation methods and recovering from surgery with the support of the mobile app-based rehabilitation programme. They said participating in the programme enabled them to undertake rehabilitation more independently.

*“I believed I could master the methods of rehabilitation with the support of the programme. … I did the rehabilitation at home in a more independent way. ... I knew as long as I followed the programme and persevered patiently, I could complete the rehabilitation programme and recover from the surgery.” (P3, male, TKA)*

The participants said that by watching exercise demonstration videos provided in the programme, they felt more confident doing exercises they were once afraid to do.

*“I used to dare not try the exercise upstairs and downstairs as I was afraid something wrong would happen. I saw the video showing that we can do it and how to do it. I remembered the movements. I was more confident to try on the stairs.” (P27, male, TKA)*

The participants said that learning from the experiences shared by previous patients who had successfully recovered from the surgery, such as addressing issues encountered during recovery, enhanced their confidence in rehabilitation self-management.

*“I liked the section about stories shared by former patients who had successfully recovered. I was particularly interested in what issues they encountered during recovery, and how they handled these issues. For example, I used to feel tightening in the skin at the surgical site. I read in the shared experiences that soaking the skin in warm water would help. I did so and it was much better…. Learning from former patients gave me the confidence to manage my condition.” (P69, male, THA)*

The participants said that they gained the confidence to practise rehabilitation exercises when they saw the videos showing that other patients who had undergone the same surgery did the exercises.

*“Sometimes I dared not to do the movements demonstrated by the demonstrators. I thought they (demonstrators) were normal people, but I was patient, could I do the movements like a normal person? Would my joint be dislocated? When I saw a patient after the same surgery did these movements, I was more confident to do exercises.” (P15, male, THA)*

The participants said the reassurance from the healthcare professionals improved their confidence in conducting rehabilitation exercises.

*“Sometimes I was afraid to do some exercise movements as I thought they might hurt my joints. I felt relieved and confident to try them after the healthcare professionals in this programme confirmed that I could do those movements.” (P15, male, THA)*

### Established supportive relationships

Eighty-four per cent (21 out of 25) participants described that the mobile app-based rehabilitation programme helped them establish supportive relationships with healthcare professionals and other patients in the programme. This included two subcategories.

#### With healthcare professionals

Many participants described that through the mobile app-based rehabilitation programme, they built a good bond with the healthcare professionals. This bond enabled the participants to share their feelings freely and get the support that allayed their worry about recovery.

*“Through talking to healthcare professionals via the app, I built a good bond with them. I regarded them as friends with whom I can talk freely. I felt relaxed sharing with them my progress and feelings, and their support eliminated my confusion and worries.” (P22, female, THA)*

#### With other patients in the programme

Most participants described the experience of establishing a supportive relationship with other patients in the programme. They said the discussion forum in the mobile programme brought them a sense of belonging, allowing them to share information, experiences, and feelings. The participants said participating in the discussion forum reduced their sense of loneliness after they were discharged from the hospital.

*“When I was discharged from the hospital, I asked other patients who had experienced recovery for suggestions. As time went by, I became an experienced patient, and I was very happy to help new patients by sharing my experiences and knowledge about rehabilitation. Here (the discussion forum via the app) we are like a big 'family'. If someone had questions or difficulties, we would like to help. ... I would have felt very lonely without the discussion forum, a place where I felt belonging to and we could support each other.” (P28, female, THA)*

The participants said that the comforting words from other patients reduced their concerns about the postoperative recovery.

*“Sometimes I was concerned about my recovery. Several experienced patients told me in the discussion forum that it was not necessary to worry too much and that everything would be fine. Their words soothed me a lot.” (P26, female, THA)*

They also described that they provided support to other patients who felt frustrated during postoperative recovery by sharing successful experiences of rehabilitation self-management.

*“If someone expressed frustration during rehabilitation in the discussion forum, I tried to comfort and encourage them. I shared how I managed the issues encountered during my recovery. I demonstrated a positive attitude towards recovery. I hoped this would help them face recovery positively.” (P22, female, THA)*

### Facilitated learning

A quarter (6 out of 25) of the participants described that the components of the mobile app-based rehabilitation programme facilitated their learning of rehabilitation instructions. It included two subcategories.

#### Facilitated understanding

The participants said the demonstration videos facilitated their learning of postoperative rehabilitation by making exercise instructions visual, interesting, and easy to understand.

*“Compared to paper-based instructions, the videos helped me to visualize the movements. Videos were more interesting and easier to understand than the instructions on paper.” (P5, male, THA)*

One participant described that although she could not read text messages due to her limited literacy competence, she felt significant improvements in recovery by practising exercises daily following the demonstration videos.

*“I am illiterate, and I do not understand text messages, but I understand the videos and pictures on the app. I did exactly what the videos show, one movement after another. I followed the demonstration videos and practised every day, and now I have improved a lot.” (P43, female, THA)*

#### Facilitated memorisation

The participants described that by engaging with exercise instructions on the app repeatedly and frequently, they could better memorise these instructions. Some participants specifically described that they felt easier to memorise exercise instructions after watching demonstration videos. They said the video demonstrations were imprinted on their mind because of visualisation.

*“Doctors taught me how to conduct exercises, but I forgot as time went by. The app programme is wonderful as I could access and learn the exercise instructions frequently and repeatedly. After several weeks I memorised most of the exercises.” (P71, female, THA)*

*“Demonstration videos are easy to visualise than the instructions on paper. They are imprinted on my mind. …It seems easier for me to memorise the exercise movements after watching the videos.” (P5, male, THA)*

### Future directions

The participants also provided suggestions regarding the future use of the mobile app-based rehabilitation programme and room for improvement. Two subcategories were revealed.

#### Future use

A quarter (6 out of 25) of the participants said that they had recommended the mobile app-based rehabilitation programme to their relatives and friends who had received similar surgeries. They suggested healthcare professionals integrate this programme into routine care in future so that more patients could access this service.

*“This programme is easy to use and quite needed by patients after arthroplasty. It should be implemented in routine care in future so that more patients can access it.” (P51, male, THA)*

Several participants suggested including family members or caregivers in the mobile app-based rehabilitation programme to provide support to the patient and maintain the patient’s enthusiasm for rehabilitation. They said this is important for the patient’s recovery.

*“I would like to suggest including family members or caregivers in the programme. Healthcare professionals could assign some tasks to the family members (or caregivers) to involve them. Family members can remind the older patients if they forget something. ... Family support is important to patients, not only for their physical recovery but also for psychological support. Patients' enthusiasm would be kept if the family could accompany them during the rehabilitation journey.” (P78, female, TKA)*

#### Suggested programme improvement

The participants also provided suggestions for improving the current programme. They suggested developing a frequently-asked-question section and regularly updating it in the mobile app-based rehabilitation programme as some common questions were repeatedly posted. They suggested a discussion schedule that would allow healthcare professionals to participate in discussions at regularly scheduled times, instead of being on standby 24/7. They said this might reduce the workload of healthcare professionals, and the programme might thus be more sustainable.

*“For some common questions, such as the symptoms that probably appear after hospital discharge, a frequently-asked-question section could be developed and regularly updated. Newly enrolled patients could read the question and answer, and this may answer part of their questions. Thus reduces the workload of healthcare professionals regarding the need for repeated answers to similar questions.” (P27, male, TKA)*

*“A discussion schedule could be made where the healthcare professionals attend the discussion forum several times a week. There is no need for healthcare professionals to be on 24/7 standby. In this way, the workload of healthcare professionals could be reduced, and the programme may be more sustainable.” (P26, female, THA)*

One participant suggested including health information and examples related to postoperative pain management in the mobile app-based rehabilitation programme. He said this would better support patients’ pain self-management after hospital discharge.

*“I would suggest including pain-related information in the app programme, such as why patients feel pain and how to properly manage it after discharge. The app can provide brief instructions on the painkillers commonly used after joint replacement and may provide some tips for pain management postoperatively. … The app can provide more examples of former patients, not only in rehabilitation exercises but also in pain management. It will help patients self-manage their pain while they are recovering at home.” (P61, male, TKA)*

Another participant suggested providing a video demonstrating how to use the programme in addition to the current oral explanation plus a take-home booklet. She said a demonstration video would be particularly helpful to those who live alone with little social support.

*“Although I had been taught how to use the mobile app programme and provided an illustrative booklet, I could not remember everything and had a bit of difficulty reading the booklet. If there was a video showing the steps of how to use the programme, it might be helpful. I live alone and have nobody to help. I could use the programme better if I had a handy video to watch and learn.” (P75, female, THA)*

## Discussion

This qualitative study evaluated a mobile app-based rehabilitation programme from the perspectives of 25 patients who had access to the programme during their rehabilitation after THA and TKA. The findings provide an in-depth understanding of patients’ perceptions of the strengths and weaknesses of the programme as well as room for improvement. The patients perceived the newly developed mobile app-based rehabilitation programme as helpful for their postoperative rehabilitation, and they recommended incorporating the programme into routine care for patients after THA and TKA. This study adds new knowledge to the field of arthroplasty telerehabilitation as it provides an authentic description of patients’ experiences of receiving rehabilitation support via a mobile app and a discussion of this knowledge as follows.

Patients in this study perceived improved access to health care, such as comprehensive and reliable rehabilitation information and support from healthcare professionals, by participating in the mobile app-based rehabilitation programme. The patients described access to rehabilitation care as convenient and flexible via a familiar app on mobile devices. They could use the programme whenever and wherever they need it without time and place restrictions. This suggests that mobile app-based programmes can overcome some limitations of synchronous telerehabilitation modes (e.g., using videoconferencing), such as the need for both patients and healthcare providers to sit in front of a high-quality screen and access the programme simultaneously [12]. These findings support that mobile apps could be an accessible and efficient means to enhance the care of patients undergoing total hip or knee arthroplasty [13]. This is important for countries like China where face-to-face rehabilitation services are inadequate in many areas due to the lack of rehabilitation facilities and qualified healthcare professionals [42].

With the support of the mobile app-based rehabilitation programme, the patients perceived being engaged, motivated, and confident in their postoperative recovery. They highlighted the programme components, such as exercise demonstration videos, the discussion forum, reminders, and successful experiences shared by other patients, in the enhancement of their engagement, motivation, and confidence in rehabilitation practice. Such findings inform future programme developers of potentially effective components that could be included in mobile rehabilitation programmes. Patients’ experiences of improved engagement and motivation may be partially attributed to the application of Bandura's self-efficacy theory [15] in the development of the mobile app-based rehabilitation programme. All four elements that may influence an individual’s confidence in their capability to complete rehabilitation were addressed in the programme, such as offering educational materials and reminders, sharing successful examples, and providing support from peers and healthcare professionals. Enhanced self-efficacy helps increase patients’ engagement and motivation in self-management, thereby improving health outcomes [43]. Our findings, as well as those of Meng et al.’s study [44], support the adoption of self-efficacy theory in arthroplasty rehabilitation programmes.

The patients reported developing a trustful relationship with healthcare professionals, which made them relaxed and reassured. This is congruent with previous evidence that patients in telerehabilitation experienced a close and strong relationship with their therapists [6, 22]. Further, patients in this study described the mobile app-based rehabilitation programme helping them establish a supportive relationship with other patients in the programme. This relationship reduced their sense of loneliness after hospital discharge, addressed their worries and concerns, and encouraged them in their postoperative recovery. Such findings have not been reported in previous studies investigating patients’ experiences with arthroplasty telerehabilitation [6, 22]. The reason might be that telerehabilitation programmes used in previous studies were based on videoconferencing that provided real-time communications between healthcare professionals and individual patients, whereas our programme was based on a social media app which allows for assembling a group of patients over a period of time. Maintaining social connectedness, such as regular contact with peers, is important for health promotion and a factor contributing to the success of digital health interventions [45, 46]. Our findings support the use of technologies, such as mobile apps, to help patients build a satisfactory therapeutic relationship with healthcare professionals and other patients, which can enhance patient-perceived social and emotional support [47].

The Illeris model for learning, which suggests facilitating individuals’ learning process by providing interesting and understandable content, reducing mental energy needed in learning, and improving interaction with the environment [16], was used in this programme to develop strategies supporting patients’ learning. The patients described the strategies as very helpful in facilitating their understanding of the rehabilitation instructions. An example was a patient who, despite her difficulties reading text messages, was able to complete all exercises by following the demonstration videos which she felt contributed to her recovery. As described by the study participants, the use of demonstration videos made the educational materials visual, interesting, and easy to understand, which may have made attending easier and thus facilitated learning [16]. The use of visuals also help memorisation by improving learners’ comprehension of the content and retention of information [48]. Accurate understanding and memorisation of instructions are the premises of correctly conducting rehabilitation exercises. Our findings thus support the adoption of the Illeris model for learning to develop a visual and easy-to-understand telerehabilitation solution for patients after arthroplasty.

Patients in this study perceived convenient and flexible access to health care by using the app with fewer requirements of transport, which enabled them to obtain timely support from healthcare professionals. This was particularly valuable for patients who were in the first few weeks after surgery when they needed more support but had less mobility. By participating in the mobile app-based rehabilitation programme, the patients established rapport with healthcare professionals and other patients, which reduced their worries, concerns, and sense of loneliness. They felt more confident and independent in performing the rehabilitation tasks. These findings imply that the mobile app-based rehabilitation programme may help to improve patients' satisfaction with life and physical and emotional well-being during postoperative recovery. As these are indicators of quality of life [49], the programme may thus contribute to improving patients' quality of life after arthroplasty. This supports previous evidence that mobile app-based rehabilitation improves health-related quality of life in patients after arthroplasty [11].

Given the benefits of the mobile app-based rehabilitation programme, patients in this study recommended incorporating the programme into routine care after arthroplasty. This implies that the patients regard the programme as helpful to others undergoing similar surgeries. In addition, the patients did not report technology-related difficulties using the mobile app-based rehabilitation programme. With an increasing number of older individuals, who are the main population receiving THA and TKA, becoming smartphone users and comfortable with digital technologies [50], there is a potential to increase the use of delivering rehabilitation programmes via mobile apps like WeChat. Nevertheless, the value of face-to-face contact between patients and healthcare professionals should not be ignored, especially when examining the operated joints and dealing with issues such as poor patellar mobility [6].

The patients provided suggestions on future use and improvement of the mobile app-based rehabilitation programme, such as involving family members or caregivers in the programme to support patients’ rehabilitation in the home setting, developing a frequently-asked-question section and a discussion schedule to reduce the workload of healthcare professionals, and including information related to pain self-management in the programme. These suggestions are considered feasible and reasonable. The needs of both healthcare providers and patients would be revisited after the trial to explore service sustainability in terms of healthcare resources. Future studies are warranted on how to effectively engage family members and caregivers in mobile rehabilitation programmes and the effect of mobile app-based rehabilitation on patients’ relationships with their family and caregivers.

A few patients described not reading the educational materials on psychological support because they did not perceive a need for this type of support. They viewed that only patients with psychological problems needed psychological support. Such findings suggest that some patients after arthroplasty may lack knowledge of or have a negative attitude towards psychological support, which could be related to low mental health literacy. A survey reported that Chinese adult citizens had low to moderate levels of mental health literacy, and older populations had the lowest level compared to youth and middle-aged [51]. Health education based on a Knowledge, Attitude, and Practice approach could be used to improve individuals’ mental health literacy, and it can be delivered via various methods including mobile apps [52, 53]. An educational module could be included in the orientation of a mobile app-based rehabilitation programme to explain the psychological support in the programme, why it is important, and what potential benefits it may bring, so that patients can better understand and utilise the psychological support in the programme.

### Limitations

Study participants were recruited from an RCT that was conducted at a tertiary hospital in eastern China. The nature of RCTs might reduce the diversity of the participants in this study. For instance, patients volunteering for the trial may be more familiar with technology and be positive about telehealth. Thus, the findings may not represent the perspectives of those who are not familiar with digital technology. The views of patients who receive healthcare services in other areas of China may not be fully represented, as the context of telehealth may vary across regions and health environments. This study relied on participants’ self-reported data on the completion of the mobile app-based rehabilitation programme. Future studies could include objective measures to assess the extent they complete the programme. Only patients who self-reported completing the mobile app-based rehabilitation programme were recruited, which may bias the results as motivated patients were more likely to be included. The study participants retrospectively described their experiences using a programme that lasted 6 weeks, which may have potential recall bias. All interviews were conducted via telephone to reduce physical contact during the pandemic, which impeded the collection of non-verbal information, such as the facial expressions of the participants.

### Implications for clinical practice

In the present study, nurses led a team of healthcare professionals in the design, implementation, and evaluation of a mobile app-based rehabilitation programme. This programme was found to improve patients’ access to rehabilitation care, engage and motivate patients in postoperative recovery, help them establish a supportive relationship, and facilitate their learning of rehabilitation instructions. These findings support nurses to take an active and expanded role in using mobile technologies to improve the quality of arthroplasty rehabilitation care. This study demonstrated the provision of rehabilitation information to patients in a supportive learning environment through an app-based programme that was based on a self-efficacy theory, a learning model, and patients’ needs. The patients perceived enhanced self-efficacy, good relationships with healthcare professionals and other patients, and emotional support. These outcomes are critical for patients’ effective self-management in postoperative recovery. The conceptual framework used in this study can be applied when developing new mobile app-based rehabilitation programmes for patients in other healthcare settings. A well-designed mobile app-based rehabilitation programme can deliver care in an accessible, convenient, flexible, and engaging way, which is valuable in maintaining continuity of care for patients discharged to non-medical settings, especially those who live in remote areas and who have reduced access to hospital services due to the COVID-19 pandemic or unavailability of services.

## Conclusions

Patient users reported positive experiences using the mobile app-based rehabilitation programme that was based on the self-efficacy theory and Illeris model for learning, and these included improving access to health care, encouraging postoperative recovery, establishing supportive relationships with healthcare professionals and other patients, and facilitating their learning of rehabilitation instructions. These experiences support the value of developing telehealth solutions to support patients’ rehabilitation after arthroplasty. Future studies are required to explore the implementation of mobile app-based rehabilitation in clinical practice, including incorporating the mobile app-based rehabilitation programme into routine care and expanding the scope of the programme to include more stakeholders, such as family members and caregivers.

## Supplementary Information

**Additional file 1.**

## Data Availability

The datasets analysed during the current study are available from the corresponding author on reasonable request.

## Abbreviations

THA: Total hip arthroplasty
TKA: Total knee arthroplasty
App: Application
RCT: Randomised controlled trial
